# Adenocarcinoma In Situ Arising from Brunner's Gland Treated by Endoscopic Mucosal Resection

**DOI:** 10.1155/2017/7916976

**Published:** 2017-04-23

**Authors:** Masaya Iwamuro, Sayo Kobayashi, Nobuya Ohara, Seiji Kawano, Yoshiro Kawahara, Hiroyuki Okada

**Affiliations:** ^1^Department of Gastroenterology and Hepatology, Okayama University Graduate School of Medicine, Dentistry and Pharmaceutical Sciences, Okayama 700-8558, Japan; ^2^Department of General Medicine, Okayama University Graduate School of Medicine, Dentistry and Pharmaceutical Sciences, Okayama 700-8558, Japan; ^3^Department of Internal Medicine, Fukuyama City Hospital, Fukuyama 721-8511, Japan; ^4^Department of Pathology, Okayama University Graduate School of Medicine, Dentistry and Pharmaceutical Sciences, Okayama 700-8558, Japan; ^5^Department of Pathology, Kagawa Rosai Hospital, Marugame 763-8502, Japan; ^6^Department of Endoscopy, Okayama University Hospital, Okayama 700-8558, Japan

## Abstract

An 86-year-old Japanese man was presented to our hospital for further investigation of duodenal adenocarcinoma. The tumor was endoscopically resected. Pathological analysis revealed coexistence of gastric foveolar metaplasia and a surrounding hyperplastic Brunner's gland, in addition to an adenocarcinoma component. Immunostaining for MUC5AC and MUC6 confirmed the diagnosis of adenocarcinoma in situ arising from Brunner's gland hyperplasia. This case suggests that although detailed preoperative evaluation is required to determine the depth of tumor invasion, endoscopic resection may be a promising option for the treatment of adenocarcinomas arising from Brunner's gland hyperplasia.

## 1. Introduction

Brunner's glands exist in the submucosa of the duodenum, typically in the duodenal bulb and the second portion of the duodenum proximal to the sphincter of Oddi. Although the role of Brunner's glands in digestion is controversial, it is hypothesized that a mucus-rich, bicarbonate-containing, alkaline secretion produced by Brunner's glands aids in neutralizing the acidic content of chyme and gastric acid, providing an alkaline milieu to optimize intestinal absorption and lubricate the intestinal walls [1, 2]. Brunner's gland hyperplasia and hamartoma are two representative lesions that are occasionally identified during esophagogastroduodenoscopic examination [1, 3–5]. It has been reported that Brunner's gland hyperplasia and hamartoma account for approximately five per cent of all duodenal masses [2, 6]. Moreover, in rare instances, adenocarcinoma may also arise from Brunner's glands [7–12].

We recently encountered a patient with a sessile polyp in the second portion of the duodenum that was successfully resected by endoscopic mucosal resection and diagnosed as adenocarcinoma in situ arising from the Brunner's gland. In this report, we focus mainly on the pathological characteristics of the adenocarcinoma and review previously reported cases of this disease.

## 2. Case Report

**Figure 1:**
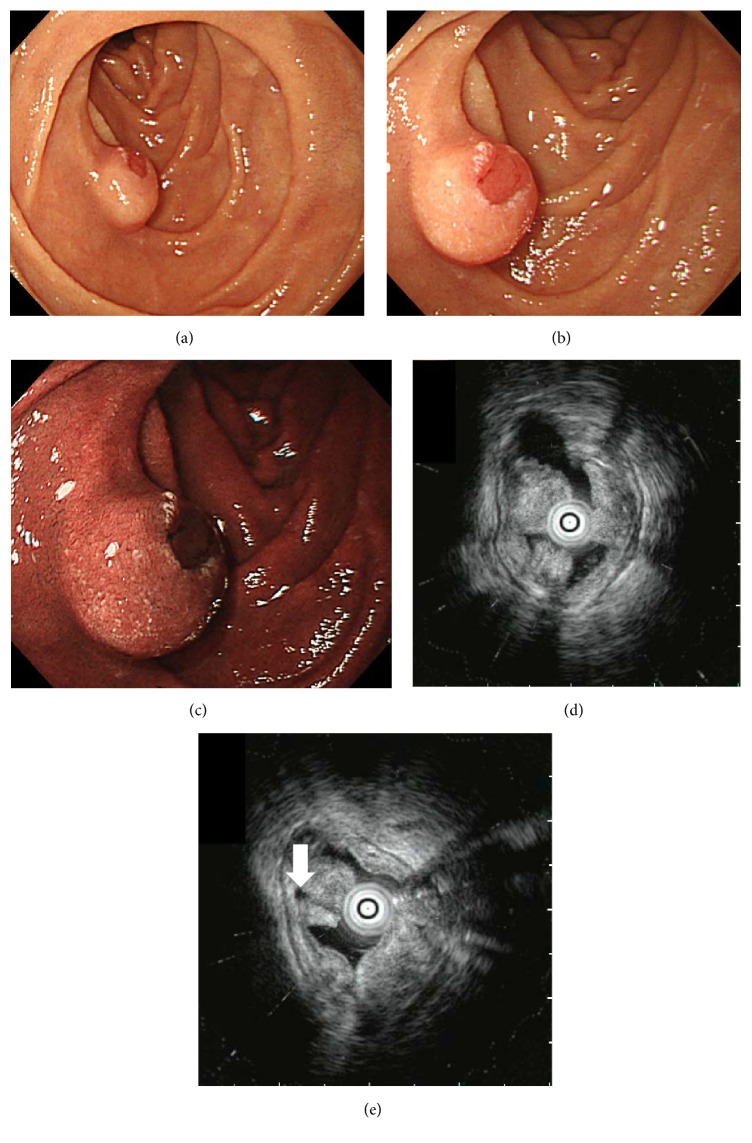
Endoscopic images. Esophagogastroduodenoscopy shows a solitary submucosal tumor in the duodenal second portion. The top of the tumor is slightly depressed, showing a reddish color on white light imaging (a, b) and a brownish color on narrow band imaging (c). Endoscopic ultrasonography reveals that the hypoechoic mass is mainly confined to the mucosal layer (d). A small cystic area is also seen (white arrow) (e).

An 86-year-old Japanese man underwent esophagogastroduodenoscopy for the investigation of anemia. An acute gastric mucosal lesion was identified in the stomach that was thought to be the cause of his anemia. In addition, a solitary submucosal tumor approximately 10 mm in diameter was found in the second portion of the duodenum (Figure 1). The top of the tumor was slightly depressed, showing a reddish color. Histological analysis of the biopsied samples obtained from the reddish part of the tumor revealed adenocarcinoma. The patient was referred to our hospital for further investigation and treatment.

The patient had been consuming medication for atrial fibrillation, diabetes mellitus, and constipation but had no history of gastroduodenal disease. A physical examination revealed conjunctival pallor and arrhythmia, but there were no abnormalities in his abdomen. Laboratory findings revealed decreased levels of red blood cells (4.03 × 10^6^/mm^3^), hemoglobin (10.6 g/dL), hematocrit (34.2%), serum iron (20 *μ*g/dL), and ferritin (29.6 ng/mL). The levels of carcinoembryonic antigen and carbohydrate antigen 19-9 were within the normal range. The patient tested positive for* Helicobacter pylori* infection.

**Figure 2:**
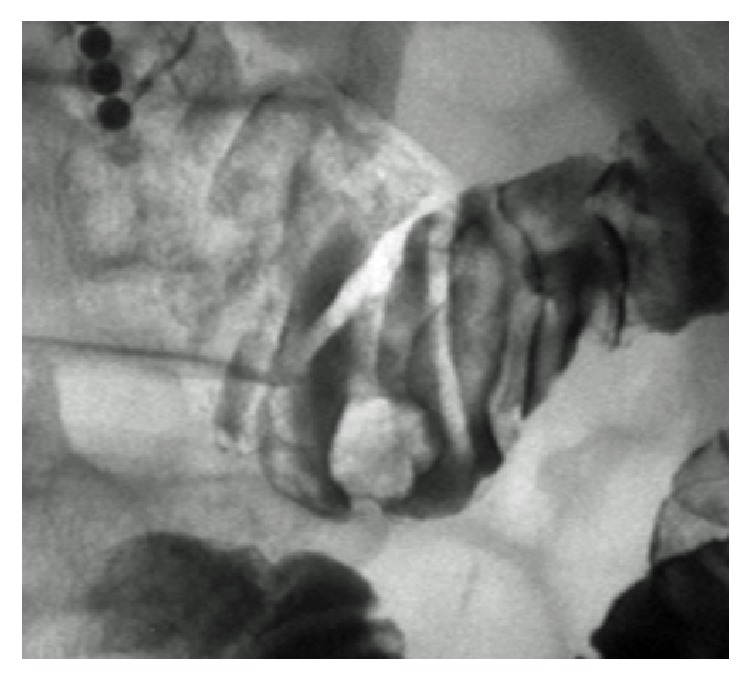
Hypotonic duodenography image. A submucosal tumor with bridging folds is seen in the inferior duodenal angle.

**Figure 3:**
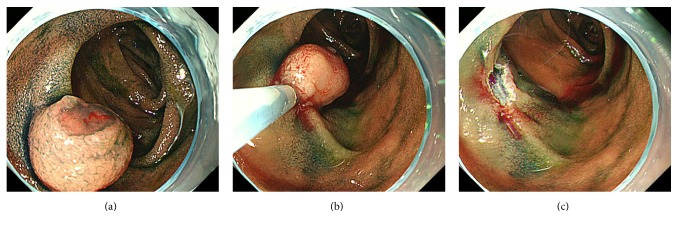
Esophagogastroduodenoscopy images during endoscopic mucosal resection. A duodenal tumor is seen after indigo carmine spraying (a). After injection of hyaluronic acid solution into the duodenal submucosa, the tumor is completely resected with a bipolar snare (b, c).

Hypotonic duodenography showed a submucosal tumor with bridging folds in the inferior duodenal angle (Figure 2). Endoscopic ultrasonography revealed a hypoechoic mass mainly confined to the mucosal layer (Figure 1(d)). A small cystic area was also identified on ultrasonography (Figure 1(e)). Based on the imaging and pathological studies, we diagnosed the duodenal tumor as adenocarcinoma arising from Brunner's glands. Due to the advanced age and multiple underlying disorders, the risks of general anesthesia, artificial ventilation, and radical surgical resection were considered too high. Moreover, endoscopic ultrasonography indicated that the tumor appeared to be resectable using the endoscopic mucosal resection technique. Therefore, we performed endoscopic treatment rather than surgical resection (Figure 3). Hyaluronic acid solution (MucoUp®, Johnson & Johnson K. K., Tokyo, Japan) was injected into the duodenal submucosa using a 23 G injection needle (TOP Co., Tokyo, Japan). Subsequently the tumor was resected with a bipolar snare (Zeon Medical Inc., Tokyo, Japan). The resected area was closed by metallic clips (Olympus Medical Systems Co., Tokyo, Japan). There were no procedure-related adverse events during or after the endoscopic resection.

**Figure 4:**
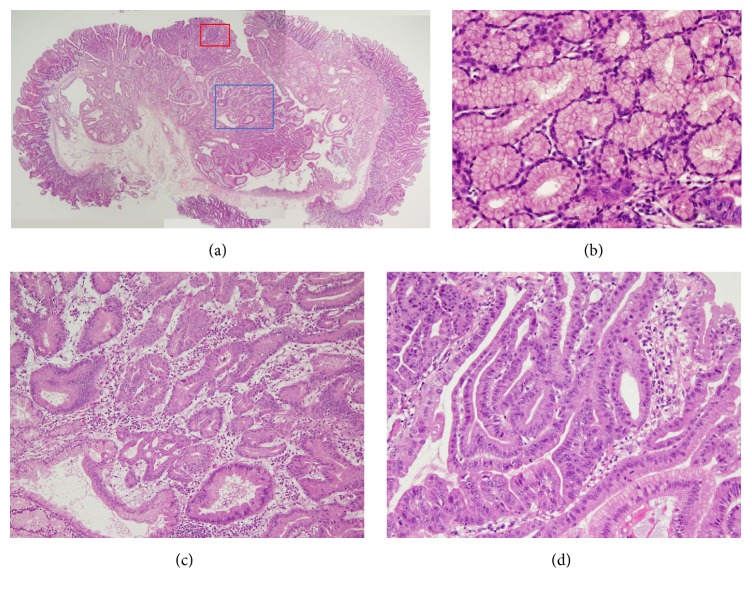
Histopathological images. Proliferation of Brunner's glands is seen in the resected specimen (a: ×2, b: ×20). Gastric foveolar metaplasia is also identified (a: blue square, c: ×10). Atypical cells with pleomorphic macronuclei with dense chromatin forming irregular glandular structure are found in the superficial layer (a: red square, d: ×20). Hematoxylin and eosin staining.

**Figure 5:**
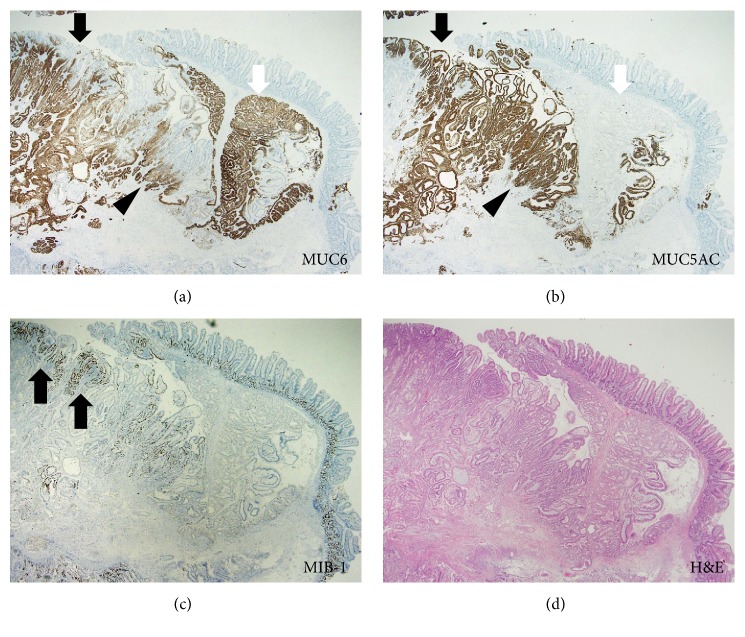
Immunohistochemical images. Proliferated Brunner's glands are positive for MUC6 (a, white arrow) and negative for MUC5AC (b, white arrow). In contrast, most of the cells with gastric foveolar differentiation (a, black arrowhead; b, black arrowhead) and atypical cells (a, black arrow; b, black arrow) are positive for MUC5AC but are negative for MUC6. Both gastric foveolar metaplasia and atypical cells partly show dual positivity for MUC5AC and MUC6, particularly in the deeper layer (a, arrowhead; b, arrowhead). Some atypical cells showed relatively higher positivity for Ki-67 staining (c, hematoxylin and eosin staining, arrows).

Pathological analysis of the resected specimen revealed proliferation of Brunner's glands (Figures 4(a) and 4(b)). Gastric foveolar metaplasia was also identified (Figure 4(a), blue square, and Figure 4(c)). Atypical cells with pleomorphic macronuclei containing dense chromatin forming an irregular glandular structure were identified towards the lumen in the superficial layer (Figure 4(a), red square, and Figure 4(d)). Immunohistochemical studies revealed that proliferated Brunner's glands were positive for MUC6 (Figure 5(a), white arrow) and negative for MUC5AC (Figure 5(b), white arrow). In contrast, most of the cells with gastric foveolar differentiation (Figures 5(a) and 5(b), arrowhead) and atypical cells (Figures 5(a) and 5(b), black arrow) were positive for MUC5AC but negative for MUC6. However, both gastric foveolar metaplasia and atypical cells partly showed dual positivity for MUC5AC and MUC6, particularly in the deeper layers (Figures 5(a) and 5(b), arrowhead). Some atypical cells showed relatively higher positivity for Ki-67 staining (Figure 5(c), arrows). Consequently, a diagnosis of adenocarcinoma in situ arising from Brunner's glands was made. Components of the adenocarcinoma, gastric foveolar metaplasia, and Brunner's gland hyperplasia were completely resected by endoscopic mucosal resection.

## 3. Discussion

Adenocarcinoma arising from Brunner's glands is quite uncommon. The first case was reported in 1894 by Pic [13]. Ohta et al. reviewed 25 previously reported cases and revealed that the mean age was 66.5 years (39 to 85 years) and the male to female ratio was 19 : 6 [14]. Adenocarcinoma arising from Brunner's glands is most frequently found in the second (50.0%) or first (45.8%) portions of the duodenum, followed by the third portion (4.2%). Macroscopically, it has been described as “a pedunculated and lobulated polyp” [11], “a protruding lesion with a surface depression” [9], “an elevated submucosal-tumor-like lesion with a shallow central depression” [9], “a sessile tumor with a slight central depression,” and “a fungating ulcerated tumor” [15]. Overall, macroscopic features of adenocarcinoma arising from Brunner's glands vary from submucosal tumor-like lesions with a shallow central depression (52.0%) to polypoid (12.0%), sessile (12.0%), and type 2 tumors (12.0%) [14]. Kamei et al. hypothesized the progression process of this disease as follows [7]: appearance as a submucosal tumor or polypoid form in the early stages with subsequent tumor growth and type 2 tumor formation. Such morphological changes during tumor progression over a 40-month period have been described by Itsuno et al. [15]. The present case showed a submucosal tumor with a shallow central depression, which is the typical morphology of early-stage adenocarcinoma arising from Brunner's glands.

In the present case, pathological analysis of the resected specimen showed three components: Brunner's gland hyperplasia, gastric foveolar metaplasia, and adenocarcinoma in situ. Although the histogenesis of gastric metaplasia in the duodenal mucosa has not been fully revealed, several studies have suggested Brunner's glands as a region where gastric metaplasia may originate [11, 16–18]. During the repair process following duodenal ulceration, gastric foveolar-type cells emerge in the regenerative cell zone in Brunner's glands next to the ulcerated surface [11]. Immunostaining analysis with gastric foveolar-type mucin (MUC5AC) and pyloric/Brunner's gland-type mucin (MUC6) demonstrated that, in the present patient, Brunner's gland hyperplastic region was positive for MUC6 and negative for MUC5AC. Conversely, the cells with gastric foveolar differentiation and adenocarcinoma were positive for MUC5AC. Both gastric foveolar metaplasia and adenocarcinoma were negative for MUC6 in the superficial layer but showed dual positivity for MUC5AC and MUC6 in the deeper layer (Figures 5(a) and 5(b)). These gradual changes in mucin expression and the seamless transition from foveolar metaplasia to adenocarcinoma components suggested that the adenocarcinoma emerged via gastric foveolar metaplasia, which originated in Brunner's gland cells. A similar expression pattern of mucin was previously described by Kitagori et al. and Kushima et al. [9, 11]. However, Kamei et al. reported a case of adenocarcinoma arising from Brunner's gland hyperplasia in which the adenocarcinoma component was negative for both MUC6 and MUC5AC [7]. The differences in the immunostaining results may be explained by the heterogeneity of cells within the adenocarcinoma between patients, or alteration of cell characteristics during tumor progression.

Adenocarcinoma arising in the duodenum is most frequently treated by pancreatoduodenectomy (36.0%), partial duodenectomy with gastrectomy (28.0%), or partial duodenectomy (16.0%) [14]. Endoscopic treatments such as endoscopic mucosal resection [12] and endoscopic polypectomy [11] have been reported in 12% of patients. In the present case, the tumor was completely resected in one piece by endoscopic mucosal resection, similar to that described in previous reports. As shown in the pathological image (Figure 4(a)), the tumor consisted mainly of hyperplastic Brunner's gland cells, rather than gastric foveolar metaplasia or adenocarcinoma components. This structure enabled curative endoscopic resection of the adenocarcinoma.

In conclusion, we treated a patient with adenocarcinoma in situ in the duodenum by endoscopic mucosal resection. Pathological analysis revealed coexistence of gastric foveolar metaplasia and surrounding hyperplastic Brunner's glands, suggesting that the adenocarcinoma arose from Brunner's gland hyperplasia. Although there is no doubt that detailed preoperative evaluation of the depth of tumor invasion is mandatory, endoscopic resection may still be a possible option for the treatment of adenocarcinoma arising from Brunner's gland hyperplasia.
